# Successes and challenges of artificial intelligence in cardiology

**DOI:** 10.3389/fdgth.2023.1201392

**Published:** 2023-06-28

**Authors:** Bert Vandenberk, Derek S. Chew, Dinesh Prasana, Sunny Gupta, Derek V. Exner

**Affiliations:** ^1^ Department of Cardiac Sciences, Libin Cardiovascular Institute, Cumming School of Medicine, University of Calgary, Calgary, AB, Canada; ^2^ Department of Cardiovascular Sciences, KU Leuven, Leuven, Belgium; ^3^ Intelense Inc., Markham, ON, Canada; ^4^ IOT/AI- Caliber Interconnect Pvt Ltd., Coimbatore, India

**Keywords:** artificial intelligence, big data, synthetic data, cardiology, electrophysiolgy

## Abstract

In the past decades there has been a substantial evolution in data management and data processing techniques. New data architectures made analysis of big data feasible, healthcare is orienting towards personalized medicine with digital health initiatives, and artificial intelligence (AI) is becoming of increasing importance. Despite being a trendy research topic, only very few applications reach the stage where they are implemented in clinical practice. This review provides an overview of current methodologies and identifies clinical and organizational challenges for AI in healthcare.

## 1. Introduction

**Figure 1:**
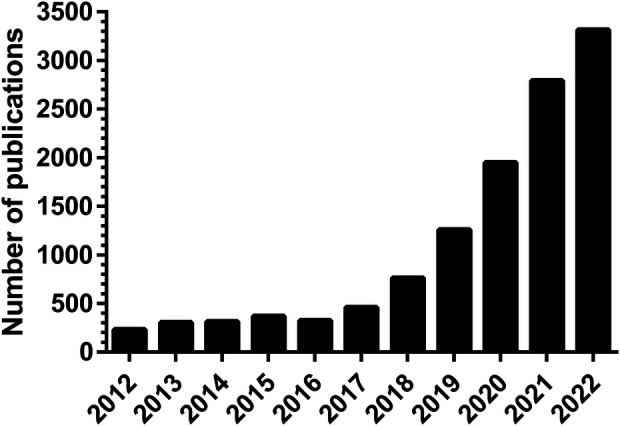
Number of publications on “artificial intelligence in healthcare” according to PubMed.

**Figure 2:**
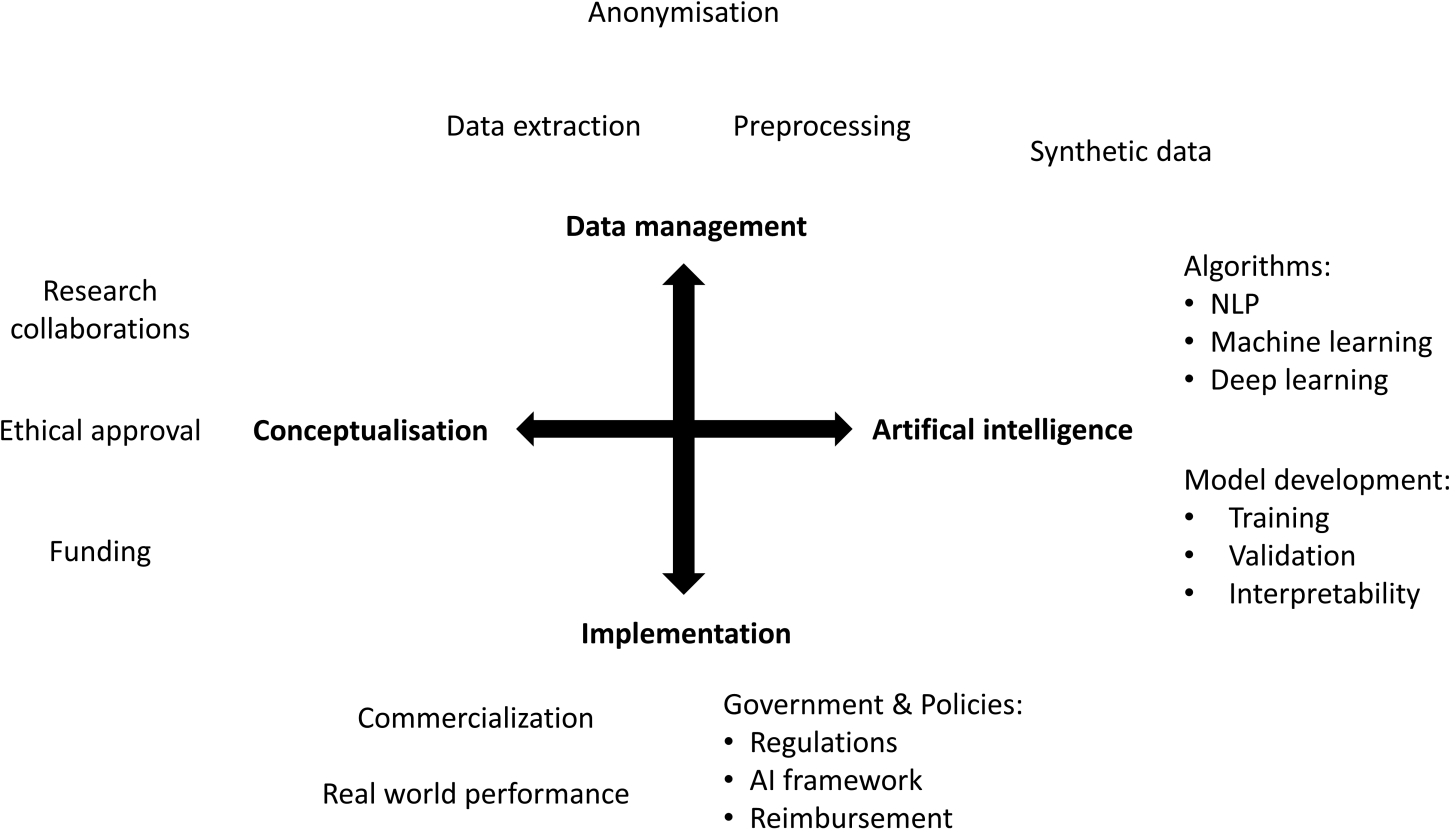
Overview of development and implementation of AI applications.

In the past decade there has been an exponential increase in the number of publications on artificial intelligence (AI) applications in healthcare (Figure 1). However, only a small proportion of these are successfully implemented in clinical practice. AI is expected to impact the entire healthcare system in the next decades, but awareness of the limitations is needed. The aim of this narrative review is to provide a comprehensive overview of current methodologies, applications, and challenges of AI in healthcare, both clinical and organizational (Figure 2).

## 2. Big data, digital health and AI

**Figure 3:**
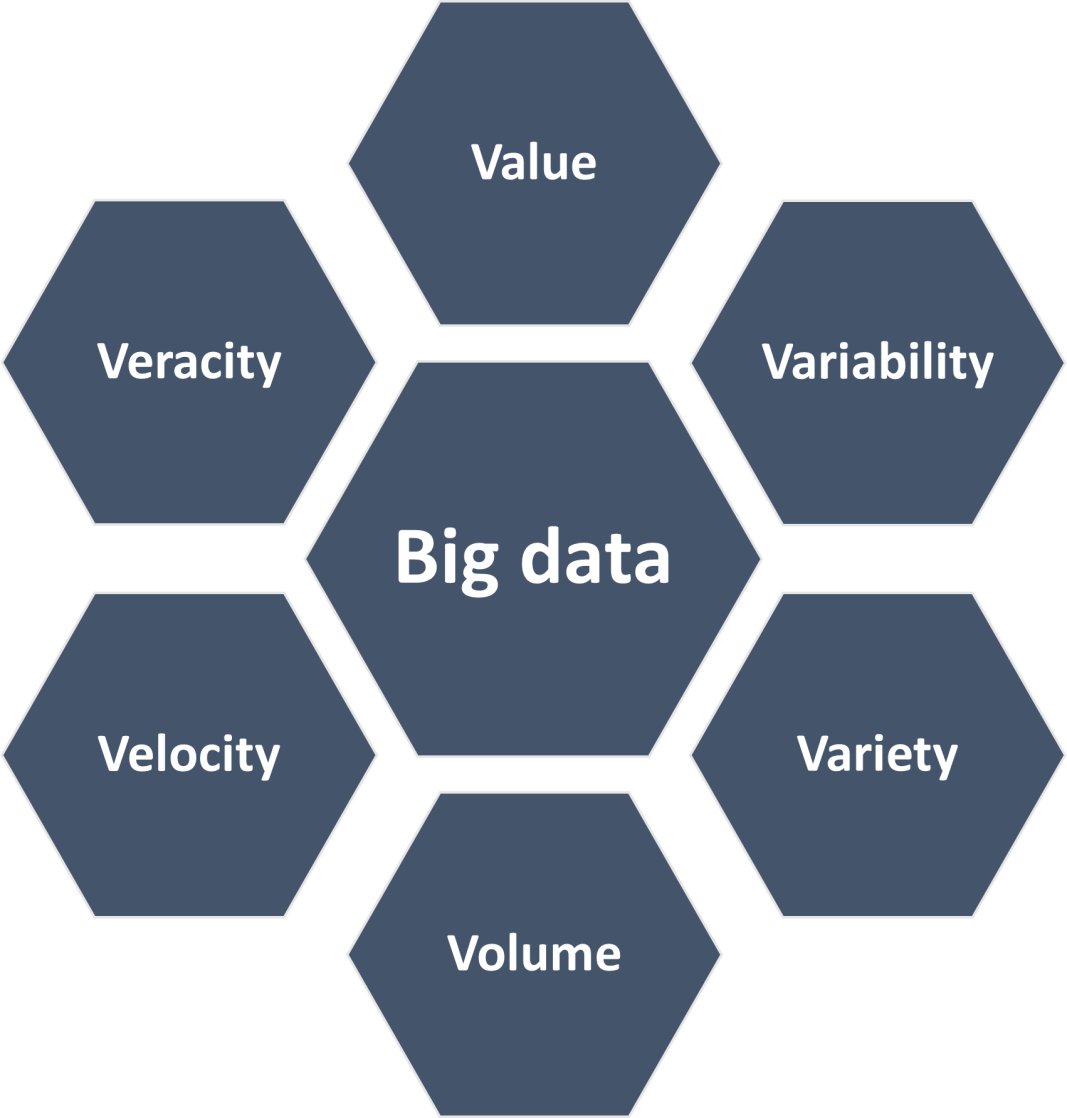
The cardinal features of big data. Variability refers to the consistency of the data over time, while variety reflects the wide range of types of data, such as images or videos. Volume refers to the magnitude of data which is generated in short time periods (velocity). The generated data yield high value, both scientific and economic, but depends on the reliability and accurateness (veracity). Adapted from Pablo et al. (2).

Healthcare has always been data-driven and with increased healthcare digitization, an overwhelming amount of data is generated. Not only from hospitals and healthcare providers, but also from other healthcare stakeholders, such as insurance and medical research. With technological advancements and the big data revolution, there is a huge potential for using this data to transform healthcare (1). Big data represents information characterized by “the 6 V's” (Figure 3), including a high volume, velocity and variety of data that require specific analysis methods to render data into value (2). Besides big data, there has been a surge in digital health applications where contemporary information and communication technologies are used to manage illnesses, health risks and to promote wellness (3). This includes wearable devices, mobile health, telehealth, and telemedicine. This evolution has the promise to improve access to healthcare, reduce inefficiencies and provide a more personalized healthcare (3).

Before AI applications can be used in healthcare, they must be “trained” using clinical or synthetic data. There is a large variety in clinical data, such as demographics, medical notes, physical examinations, and clinical laboratory results. In the past, the AI literature has mainly focused on data from diagnostic imaging, genetic testing, and electrocardiograms, whereas data from monitoring, mass screening initiatives and administrative data have been less popular (4). Along with the emergence of advanced analytics, machine learning, and artificial intelligence techniques, there are numerous possibilities for transforming this data into meaningful and actionable results. Healthcare stakeholders can use analytical techniques to harness the power of data not only for analyzing historical data (descriptive analytics), but also for predicting future outcomes (predictive analytics) and determining the best action for the current situation (prescriptive analytics) (1).

Despite the wide availability of clinical data, there is a need for more precise and focused data which can be achieved by generating synthetic data. Synthetic data refers to any production data applicable to a given situation that is not obtained by direct measurement, but generated to meet specific needs or conditions (5). The generation of realistic, synthetic, behavior-based sensor data is a critical step in testing machine learning techniques for healthcare applications. Many existing methods to generate synthetic data are limited in complexity and realism. One of the preferred approaches is to use hidden Markov and regression models that are initially trained on real datasets to generate synthetic time series data composed of nested sequences (6). Time series distance measures can be used as a baseline to assess how realistic the synthetic data is in comparison to real data. It has been shown that this produces more realistic data when compared to random data generation, data from another device, and data from another time period (6). Even in the problem of limited available real data, synthetic data methods have shown sufficient reliability to be used in real world machine learning applications (6).

## 3. Technical overview of common AI methods in healthcare, focused on cardiology

**Figure 4:**
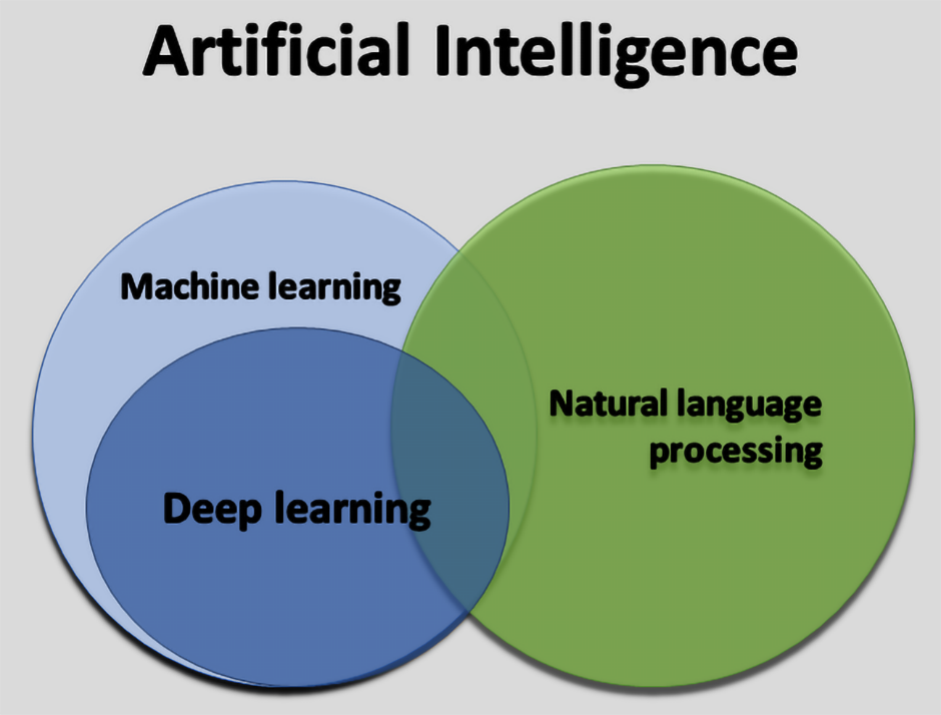
The relation between artificial intelligence, machine learning, deep learning, and natural language processing.

AI can be defined as the ability of a computer to complete tasks in a manner typically associated with a rational human being (Figure 4) (7). Machine learning (ML) is the ability for AI systems to acquire their own knowledge by extracting patterns from labelled data (supervised learning) or raw data without labels (unsupervised learning) (7). Deep learning is a machine learning method in which neural networks are created to mimic the functionality of a human neural system (7). Lastly, natural language processing is an area within AI that applies ML and deep learning techniques, among others, to analyze, interpret and transform text (7).

### 3.1. Machine learning

**Figure 5:**
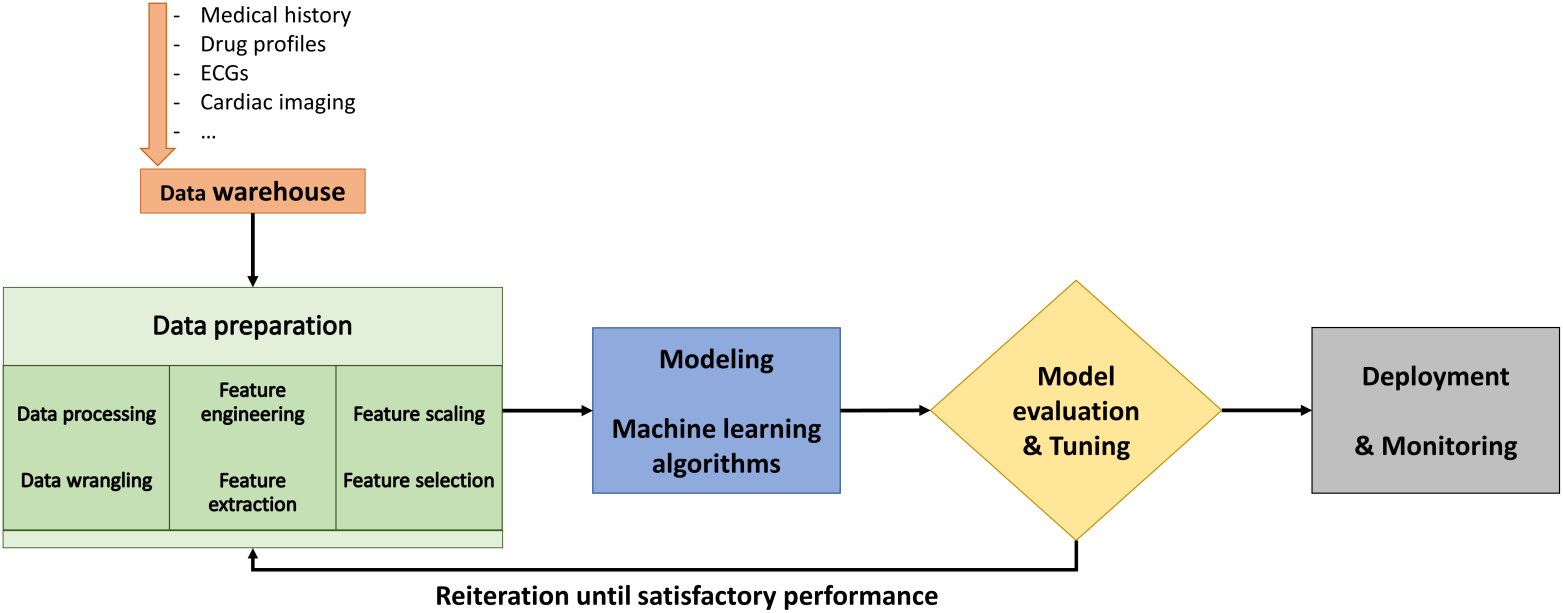
Schematic overview of a typical workflow in machine learning.

The objective of ML is to train a model that relates input data, referred to as features, to labeled outcomes (8, 9). The typical workflow in ML is presented in Figure 5. Traditional supervised ML includes training the model by learning relationships between data features and the labels. The models are based on supervised ML classification algorithms, which are capable of learning linear and nonlinear relationships (7). However, the most critical component to the performance of a model is the feature engineering and selection. As it is unpredictable which algorithm will give the best results, model development is an empiric process that involves algorithm selection and hyperparameter adjustment. Hyperparameters are parameters defined by the user to control the learning process which should be finetuned during the learning process. Lastly, the evaluation of a model's performance, including assessment for bias and overfitting, is dependent on the extent of the available data after partitioning into training, validation, and testing sets (10).

In contrast, unsupervised ML does not use the traditional training approach, but rather tries to find an underlying structure or natural patterns in the data (11). Many of these unsupervised ML models are based on cluster analysis and dimensionality reduction. Dimensionality reduction includes techniques that reduce high-dimensional data into lower-dimensional representations but preserving relevant variations and structure. Therefore, these techniques facilitate cluster analysis. Cluster analysis tries to find subgroups withing complex data, by for example hierarchical or k-means clustering (11).

### 3.2. Deep learning

In contrast to ML, deep learning (DL) is capable of automatically computing and selecting relevant features from raw input data. As DL does not require manual feature engineering, the actual strength of DL is its freedom and flexibility to use the raw input data in the most potent way possible. DL models are based on artificial neural networks, which represents computational systems that are designed like biological neuronal connections. The input data is transformed by a layer of nodes, which represent neurons, to represent the data and ultimately connects the input data with the labeled output. The nodes are organized in layers, with either fully connected layers or hidden layers. The latter are the layers between the input and output layer. The connections between the nodes represent neuronal synapses and are quantified by weights. The success of DL can be explained by the fact that in these advanced neuronal networks the layers do not need to be fully connected, as in not all nodes of one layer must be connected to the next layer, as well as the use of many successive hidden layers (7). In cardiology DL often involves the use of deep convolutional neural networks (CNN) which predict a categorical outcome based on raw input data, such as echocardiography images or electrocardiograms (12). In CNNs the building blocks of the DL model are convolutional layers where each layer uses as set of mathematical filters that detect data features to construct feature maps. Each convolutional layer is followed by a pooling layer creating feature maps. By repeating convolutional layers and feature maps, a hierarchical representation of the data is created, which can learn how particular shapes add up to complex representations and eventually generates global data classifications. Due to their high processing and mathematical potential, CNNs are the standard models in recent research (13, 14). Further advances have resulted in the ability to process data in time by adding a temporal sequence to a recurrent neuronal network and the use of transfer learning where layers and weights from a previous trained model form the basis for a new model with a different, but slightly related task (15).

### 3.3. Interpretability

In medicine, the black box nature of DL is considered a major limitation as it limits the interpretability of the feature selection process and relative weights (16). Interpretability is defined as the extent to which a human can understand the model, which includes it being unambiguous and not too complex (17). Lack of interpretability and transparency has been identified as one the main barriers to implementation of AI in clinical practice (18). Many different methods to achieve interpretability have been described. On one hand there is explainable modelling, where the internal functioning of the AI model is open and accessible to the user (17). However, explainable modeling comes with a trade-off between interpretability and model performance. On the other hand, one can use *post-hoc* explanations, either model-agnostic applicable to any type of model or model-specific, to render an AI model partly explainable without opening the black box. Overall, 3 classes of explanations are available (17).

• Model-based: A separate model is used to explain the AI model. This can either be applied as explainable modelling or as a *post-hoc* method when a more interpretable, surrogate model is created of the full AI model.
• Attribution-based: The explanatory power of each feature is ranked, measured and/or visualized. The majority of *post-hoc* explanations methods are considered attribution-based explanations.
• Example-based: A selected part of the dataset is used to explain the model by, for example, illustrating both excellent and poor predictions.

Overall, interpretability of AI models is limited by several factors. First, explainability of AI models is influenced by the comprehensibility of input features. Second, in case of *post-hoc* explanations the black box remains closed and there is no ground truth available for comparison. In healthcare explainable modelling has advantages over *post-hoc* explanations as the first goal is to create trustworthy AI models ready for implementation. However, DL with *post-hoc* explanation may be more powerful, even though the usefulness of interpretable DL models may be limited (16).

### 3.4. Natural language processing

Natural language processing (NLP) dates to the 1950s at the intersection of linguistics and AI. Initially, NLP was focused on standard parsing approaches. As the goal of NLP evolved to extract meaning from text which would be eligible for further analysis, a more statistical approach was required. This task is further complicated by the desire to extract causal relationships, temporal inferences and true information extraction (19). NLP uses common supervised or unsupervised ML methods, such as support vector machines, hidden Markov models, conditional random fields or N-grams (19). Each of these result in a specific structure and may be used in particular situations. NLP has a huge potential to facilitate clinical and research activities. Vaid et al. illustrated this potential by using NLP to extract non-numerical data from unstructured echocardiography reports (20). Using an iteratively expending rule-based approach they captured right ventricular function and valvular disease severity with an overall accuracy of 99.7% (20).

## 4. Clinical applications of AI in healthcare

Currently, clinical AI applications in healthcare are focused on 5 main domains. To provide a practical overview, these 5 domains are explored using recent AI applications.

### 4.1. Diagnostic applications

AI applications are improving the diagnostic potential of known and newer technologies, such as wearables and mobile health. Some of the best examples of the potential of AI is in the detection of atrial fibrillation based on wearable technologies. Atrial fibrillation screening in asymptomatic subjects using photoplethysmography (PPG) technology in wristbands and watches paired with AI machine learning yielded a positive predictive value of 92% (21). While an electrocardiogram with visual interpretation is a poor screening test for left ventricular dysfunction, a convolutional neural network trained on paired echocardiogram and electrocardiogram data had an AUC of 0.93 and is currently being tested in a prospective randomized clinical trial (13, 22).

### 4.2. Imaging and data visualization

In the last decade, diagnostic imaging studies such as magnetic resonance (MR), have achieved a major role in medicine. Analyzing cardiac MR data includes time-consuming and error-prone labor, such as tissue fibrosis quantification, and atrial or ventricular segmentation. However, deep learning techniques have shown to be useful for image processing and segmentation, reducing the processing time and inter-observer variability (23).

A different approach is using clinical data, such as electrocardiograms and echocardiograms, to train machine learning algorithms or deep neural networks to detect and visualize disease-specific features. Recently, EchoNet-Labs, a video-based deep learning algorithm, was presented. Using routine apical 4-chamber 2D videos, the application was able to detect anemia, elevated brain natriuretic peptide and elevated troponin I (24).

### 4.3. Clinical decision support applications

Clinical decision support systems (CDSS) combine patient information and evidence-based medicine to improve healthcare delivery by enhancing medical decisions. They can be used to successfully implement clinical guidelines, such as the Lucia Atrial Fibrillation Application which combines improved electrocardiogram-based diagnosis of atrial fibrillation with the calculation of CHA_2_DS_2_-VASc (congestive heart failure, hypertension, age ≥75 (doubled), diabetes, stroke (doubled), vascular disease, age 65–74, and female sex) and HAS-BLED (hypertension, abnormal liver/renal function, stroke history, bleeding history or predisposition, labile INR, elderly, drug/alcohol usage) scores, to support the decision for guideline-recommended anticoagulation (25, 26). CDSS can also deliver evidence-based support in differential diagnoses or clinical management. For example, the MISSION Syncope application (Multilevel Implementation Strategy for Syncope optImal care thrOugh eNgagement) integrates patient information with clinical findings to provide an evidence-based differential diagnosis, a prognosis and recommendations based on current clinical guidelines (27).

### 4.4. Novel characterization of diseases for precision medicine

In 2015 the Precision Medicine Initiative (PMI) was launched in order to develop an innovative healthcare model which tailors disease prevention and treatment at a patient level while incorporating variability in environments, genetics, and lifestyles (28). Therefore, precision medicine requires AI models incorporating available data from individual patients, preferably with interpretability of the models to improve our understanding of the disease. The added value of AI goes beyond routine clinical interpretation.

Using coronary CT angiography, a machine learning method was able to significantly improve the prediction of major adverse cardiac events beyond traditional risk factors by profiling the perivascular adipose tissue (29). In this study 167 patients scheduled for cardiac surgery underwent coronary CT angiography. During surgery epicardial fat biopsies were performed, which were investigated for inflammation, fibrosis, and vascularity. Subsequently, the radiographic signature of the epicardial fat was extracted using 843 radiomic features expressing, amongst others, the shape and texture of the epicardial fat, and correlated these with the biopsy findings. In a subsequent case-control study, a random forest machine learning model was trained to predict major adverse cardiovascular events and subsequently validated in an independent study cohort reaching a C-statistic of 0.77 (95% CI 0.62–0.93) (29).

Traditionally, predicting clinical responders to cardiac resynchronization therapy beyond the guideline-directed indications of bundle branch block and QRS duration, has been challenging. A machine learning model on the COMPANION study (Comparison of Medical Therapy, Pacing, and Defibrillation in Heart Failure trial) data using 45 commonly available baseline variables improved differentiation of patients with all-cause mortality (30, 31). While the conventional model failed to predict all-cause mortality (logistic regression AUC 0.67, 95% CI 0.65–0.69), the random forest model reached an AUC of 0.74 (95% CI 0.72–0.76) (30).

### 4.5. Characterization of rare diseases

Rare diseases are defined as disease with a prevalence less than 1 in 2,000, and currently over 7,000 rare diseases are defined worldwide (32). Approximately 80% of these have a genetic etiology and about 75% affect children. In genome-wide association studies, noncoding regions account for approximately 90% of causal disease loci. This suggests that penetrant noncoding variants and multilocus genetic patterns may be the underlying substrate in rare diseases. The use of AI applications has shown to improve the detection of these patterns. For example, SpliceAI (Illumina 2019, Cambridge, MA), a deep residual neural network, identified a pathogenic variant in the MYBPC3 intron associated with hypertrophic cardiomyopathy (33).

In general, deep learning models require large amounts of data for training and validation, which is rarely available in rare diseases. In such cases, transfer learning might be an option to work on smaller datasets. Bleijendaal et al. reported on 155 patients with phospholamban mutations and 155 age- and sex-matched controls (34). First, they found that machine learning and deep learning models trained on the available data outperformed the experts, who were only presented the anonymized ECG, with regards to accuracy and AUC (34). In a second study, they showed that the performance of the deep learning models could be increased by transfer learning (15). Using a prior deep learning model to identify sex trained on 256 278 ECGs, and the same dataset described in their first study, they were able to further improve the performance of the model significantly (AUC 0.87 vs. 0.71) (15). Similar findings have been reported for diagnosing long QT syndrome (14).

### 4.6. Drug discovery

Drug discovery is a costly and time-consuming process, but over 90% of drug molecules fail to pass phase II trials (35). AI applications have the potential to facilitate drug repositioning and development of new drugs, by simulating drug properties and activity prediction, which is of particular interest in rare diseases. A first potential for AI is the drug-target interaction modeling which involves 3-dimensenional modeling of the target (36). A second potential for AI is prediction of side effects, such as QT prolongation by human ether-à-go-go-related gene (hERG) potassium-channel blocking effects (37, 38). Siramshetty et al. predicted hERG effects of over 9,000 compounds using a random Forest machine learning model and compared the results with a deep neural network (37). They found that in a prospective validation set the deep neural network slightly outperformed the machine learning models when predicting the hERG effect of new compounds (37).

## 5. Organizational applications of AI in healthcare

AI has the potential to be transformative regarding healthcare delivery and the organizational management processes. Specifically, AI may facilitate better patient outcomes, and improve the efficiency of care delivery through augmenting and automating AI applications (39). Given the data access required to power AI algorithms, the safety and privacy of the patients will need to be protected at all costs, which will present challenges for deployment of AI within health systems. Block chain technology, well known from the bitcoin era, may provide solid and reliable data safety mechanisms. This section includes an overview of (a) optimization of health system processes and (b) concerns related to data safety and security.

### 5.1. Health system management and process optimization

The full potential of AI and its impact on the organizational processes within healthcare have yet to be realized. However, early adoption is likely targeted at addressing routine and repetitive tasks that improve clinician workflow. For example, like preliminary computer-based interpretations that accompany 12-lead electrocardiograms, AI-facilitated cardiac imaging interpretation, as described in Section 4.2, may allow for enhanced throughput, faster interpretation without compromising quality, and the optimization of repetitive tasks such as reporting. Although these AI applications are targeted at the clinician-level, health system benefits may include improved organization efficiency, reduced costs, and potentially improved clinical outcomes.

Other clinical applications with potential impact at the health system level include AI-powered risk stratification and clinical triaging. For example, Adedinsewo and colleagues validated an AI-enabled ECG algorithm to identify severe left ventricular (LV) systolic dysfunction (defined as an LV ejection fraction ≤35%) among patients presenting to the emergency department with the common clinical symptom of dyspnea (40). Patients with left ventricular systolic dysfunction may require frequent health care resources in the future, such as emergency department visits or hospitalization for decompensated heart failure (41, 42). Earlier identification and initiation of goal directed medical therapy may alter the disease course and prevent future burden on the health care system.

Another potentially disruptive algorithm includes a ML software produced by the company Corti (Corti.ai, Denmark). Corti's software is designed to “listen” into emergency responder calls and facilitate clinical-decision making by emergency dispatchers by improving time of recognition of impeding out-of-hospital cardiac arrests through voice analysis of tone, breathing patterns and other metadata. In a training and validation study using 108,607 calls to the Emergency Medical Dispatch Center of Copenhagen in 2014, time-to-recognition was shorter using Corti's algorithm compared to traditional dispatch (43). Corti has subsequently partnered with the European Emergency Number Association for deployment of the algorithm at two pilot sites in Italy and France (44).

Despite the promise of improved healthcare processes and improved patient care, there remain concerns regarding the unintended consequences of large-scale deployment of AI technologies. For example, deployment of wearables that detect atrial fibrillation led to concerns with over-diagnosis and unnecessarily subsequent health resource use and testing due to false positive readings (45). Ongoing assessment of health system impacts following AI deployment will be critical to ensure successful implementation into clinical health systems.

### 5.2. Security and data safety

A substantial barrier to AI deployment within health systems is the concern related to data privacy and security. That is, AI potentially may require integration of multiple data elements (i.e., descriptors from EHRs, wearable devices, genetic information, etc.) in large central repositories for data analysis. Traditional large clinical databases, particularly those that aggregate big data, face growing security challenges. Blockchain technologies, pioneered by the financial sector, may help address these concerns given the prerequisite data storage and access necessary for clinical AI applications (46).

While a distinct concept from AI (i.e., data analytics), blockchain technology ensures that data is valid, secure, and decentralized. The primary difference between traditional databases and the blockchain is how the data is structured, where the blockchain is a system of recording information that is decentralized. It is a digital ledger of transactions (grouped into blocks) that are duplicated and distributed across a network of computers on the blockchain. The integrity and data security of the block of information is assured cryptographic algorithms known as hash functions (47). However, blockchains are not infallible regarding security. While difficult to hack, a potential weakness is the “51% Attack” where blockchain miners control over 50% of the network's computer power, thus circumventing the data security assured through decentralization of data; individuals have stolen almost $2 billion in cryptocurrencies by exploiting this weakness (48, 49). However, the risk of a successful “51% Attack” is believed to be low given the computing power and coordination required to take over the hash power of adequately-sized blockchains (47).

Nevertheless, as AI deployment within health care systems becomes increasingly frequent, blockchain technology will become an important part of health care infrastructure. One implementation example is MedRec (2018, Cambridge, MA), which was developed in partnership between Beth Israel Deaconess Medical Center and Massachusetts Institute of Technology (MIT) Media Lab (46). The MedRec platform leverages blockchain technology to authorize and manage data sharing between healthcare systems in a decentralized approach. That is, while audit logs are maintained in the blockchain itself, identifiable health care information is not stored in the blockchain and retained in the source EHR systems (50).

In summary, blockchains (a) may provide high levels of data security through its unique data storage patterns, (b) can maintain the fidelity of health care data due to the difficulty in manipulating the blockchain itself, and (c) could facilitate accountability and authentication for data access (51). Alternative security and data protection measures are likely required but should function as part of an overarching framework.

## 6. Implementation of AI into clinical care models

The future of AI faces many challenges when implementing AI applications into daily clinical routine and patient care. Clinical care informed by AI will require a new economic framework in healthcare, taking into account the costs and liability of AI applications. Additionally, ethical concerns have been raised regarding how and when AI should be applied (52). This section includes an overview of (a) funding models and reimbursement processes for AI applications, (b) assessing the value proposition of AI within healthcare and (c) ethical considerations for AI implementation.

### 6.1. Healthcare funding models to facilitate uptake of AI

In value-based payment models, where it becomes increasingly important to improve quality care and efficiency at decreased operating costs, AI becomes a valuable tool for health care systems. It is unsurprising that health systems that historically have attempted to implement value-based care programs are now assessing reimbursement models for AI. For example, the Centers for Medicare and Medicaid Services in the United States recently established payment codes for specific AI tools for diagnosis of diabetic retinopathy, IDx-RX (Digital Diagnostics, Coralville, IA) and the use of Viz.ai software (2016, San Francisco, CA), which facilitates the diagnosis management of ischemic stroke (53).

In the United Kingdom, the National Health Service (NHS) of England has implemented the use of an AI-power FFRCT Analysis (HeartFlow, Inc.). The HeartFlow Analysis is applied to CT coronary images and uses AI technology to assess severity of stenoses by simulating blood flow and providing estimated fractional flow rates, which is a marker of severity used in invasive coronary angiography. This information facilitates clinician-decision making whether a coronary blockage is severe enough to warrant invasive management (i.e., FFRCT values ≤0.80) (54, 55). The NHS mandate for implementation of the HeartFlow Analysis at the health system level was based on an economic analysis by the National Institute of Clinical Excellence (NICE), the UK national health technology assessment body. NICE found that the HeartFlow Analysis could save NHS England approximately £391 per patient by reducing unnecessary invasive diagnostic procedures and cutting hospital waiting times (56).

Funding models for clinical applications are in their infancy. However, well-defined criteria for potential reimbursement of AI tools and applications will be critical for wide-scale adoption. Creative funding models may need to be developed to promote enthusiasm at both the health care provider level and health care organization level. For example, HeartFlow was adopted as one of four initial technologies through a special NHS MedTech Funding Mandate policy designed to improve uptake of selected innovative medical devices, diagnostics and digital products (57).

### 6.2. Evaluating the healthcare value of AI

As a strategic investment, AI may require reconfiguration of existing clinical care models or development of new models of health service delivery. Implementation may be expensive such as the potential added human resource costs of personnel re-training in addition to the initial costs of deploying AI into clinical practice. Since healthcare spending continues to consume an increasing proportion of national gross domestic products, it is increasingly important that new health technologies demonstrate health care “value”. That is in the context of finite health resources, assessment of value weighs the incremental costs associated with a new technology by the potential clinical benefits, such as improvement in quality of life or increased life expectancies (58). These costs are then compared to societal thresholds for health care value. However, there are few available economic evaluations of cardiovascular AI technologies (59). A notable exception is the early adoption of HeartFlow within NHS England, driven by NICE health technology assessment as previously described.

The accelerated development and iterative nature of AI technology may require novel frameworks for assessing health care value compared to traditional health technology assessment. For example, NICE recently a comprehensive health technology assessment framework specific to AI and other digital health technologies, which provides recommendations on the level and quality of assessment of these technologies (60). Similar digital health and AI frameworks for health technology assessment have been developed by France, Germany, Finland and South Korea (59, 61).

### 6.3. Ethical considerations

Implementation of AI implies continuous data collection of health information in centralized databases to facilitate AI analyses. This requirement raises ethical concerns regarding data ownership (i.e., the patient as an owner of their data, private sector as a technology vendor, or the health system responsible for technology reimbursement), scope of data use, safety and algorithmic fairness and bias.

As previously described, any ML-algorithm is only as effective as the data it was trained with. In fields such as healthcare, where clinical AI-power tools directly impact patient safety, accuracy of AI algorithms is critical, and proper validation with high-quality datasets is essential (62). The source of the training dataset also has implications with regards to AI bias and discrimination. For example, a disease-detection algorithms trained on datasets derived from primarily Caucasian populations, may provide less accurate or potentially inaccurate diagnosis for other populations, where training and validation data was underinclusive (63). Thus, without concerted attempts to improve inclusiveness, through training on datasets representative across race/ethnicity, gender and socioeconomic status, ML- and AI-technologies may potentially exacerbate existing health inequities (64).

Data privacy and confidentially issues have been previously discussed in Section 5.2. Another ethical consideration surrounds patient informed consent when integrating AI into clinical practice. That is, what are the circumstances where informed consent is required, and what are the responsibilities of the clinician to educate patients around AI? Specifically, do clinicians have a responsibility to discuss the type of ML-algorithm used and short-comings or potential biases of the data used analogous to describing the risks, benefits, and operation characteristics of cardiac procedures such as coronary angiography? Although aimed to improve data protection rules in a “digital age”, the European Union's General Data Protection Regulation (GDPR) have highlighted the complexities of decision making through AI and black-box algorithms that defy human understanding (65). With requirements for the provision of “meaningful information about the logic involved as well as the significance and the envisaged consequences of such processing for the data subject”, it is currently unclear how regulatory frameworks such as GDPR will interact with the implementation of AI tools in healthcare (66).

## 7. Regulatory approval and commercialization of AI in healthcare

The processes related to market approval of AI products in healthcare are evolving. The Food and Drug Administration (FDA) released the AI/ML-Based Software as a Medical Device (SaMD) Action Plan in 2021 in response to feedback from stakeholders (67). This document discusses a variety of issues, including a tailored regulatory framework for AI/ML-based SaMD, Good Machine Learning Practice (GMLP), patient-centered approach incorporating transparency to users, regulatory science methods related to algorithm bias and robustness, and real world performance. The GMLP describes a set of best practices for AI/ML models (e.g., data management, feature extraction, training, interpretability, evaluation and documentation) that are akin to good software engineering practices or quality system practices. Development and adoption of these practices is important not only for guiding the industry and product development, but also for facilitating oversight of these complex products, through manufacturer's adherence to well established best practices and/or standards. There have been many efforts to date to describe standards and best practices that could comprise GMLP, including those mentioned below. Stakeholders generally provided strong support for the idea and importance of GMLP. Additionally, there was a request for FDA to encourage harmonization of the numerous efforts to develop GMLP, including through consensus standards efforts, leveraging already existing workstreams, and involvement of other communities focused on AI/ML.

## 8. Future directions of AI in healthcare

In the next decades, AI has the potential to provide us with scientific discoveries, improved risk prediction models and amelioration of health care system processes. However, this will require additional funding and organizational frameworks to guide AI applications from development to successful implementation (Figure 3). Future clinical innovations by AI depend on extensive collaborations between medicine and engineering departments, with an important added value for interpretability to enhance knowledge, understanding and scientific progress. To achieve the full potential of AI, data collection and access are of major importance. Generalizability of AI applications depend on the quality and representativeness of the available data on which AI models are trained. To manage all this efficiently, and successfully implement novel AI applications in clinical practice, multilevel frameworks are required with a focus on patient privacy, and data safety, regulatory approval, and reimbursement.

## 9. Conclusions

AI is a fast-growing, multi-disciplinary part of healthcare with the potential to alter biomedical research, clinical practice, and healthcare organization significantly. Translating and deployment of AI applications into clinical reality remains challenging, illustrating the need for a structural framework to facilitate the entire process from the beginning. The healthcare professionals of the future should gain knowledge in AI to interpret the results, understand the limitations and adopt promising applications into clinical practice.
